# Supplementary material for: The Effect of Laminaria japonica on Metabolic Syndrome: A Systematic Review of Its Efficacy and Mechanism of Action
Source: Nutrients. 2022 Jul 25;14(15):3046. doi: 10.3390/nu14153046 (PMC9370431; doi:10.3390/nu14153046)
Supplement: Supplementary file 1 [file nutrients-14-03046-s001.zip › nutrients-1793208-Supplementary.pdf]

Supplementary Table S1. Database search strategies.

Database	Search strategy	Date	Results
**PubMed**	(((randomized controlled trial) OR (rct)) OR (clinical trial)) AND ((((laminaria japonica) OR (kelp)) OR (kombu)) OR (kunbu))	2021.08.24	145
**Web of Science**	(TS=("Laminaria japonica" OR kelp OR kombu OR kunbu)) AND (TS=(Metabolic OR Reaven OR "Syndrome X" OR Cardiovascular OR hypertension OR HTN OR blood pressure OR Glucose OR diabetes OR hyperglycemia OR Insulin resistance OR Triglyceride OR HDL OR Cholesterol OR dyslipidemia OR Waist circumference OR fat OR overweight OR body weight OR obes*))	2021.09.27	396
**EMBASE**	('laminaria japonica':ab,ti OR kelp:ab,ti OR kombu:ab,ti OR kunbu:ab,ti) AND (metabolic:ab,ti OR reaven:ab,ti OR 'syndrome x':ab,ti OR cardiovascular:ab,ti OR hypertension:ab,ti OR htn:ab,ti OR 'blood pressure':ab,ti OR glucose:ab,ti OR diabetes:ab,ti OR hyperglycemia:ab,ti OR 'insulin resistance':ab,ti OR triglyceride:ab,ti OR hdl:ab,ti OR cholesterol:ab,ti OR dyslipidemia:ab,ti OR 'waist circumference':ab,ti OR fat:ab,ti OR overweight:ab,ti OR 'body weight':ab,ti OR obes*:ab,ti)	2021.09.15	168
**Cochrane Central Register of Controlled Trials Library**	#1. Laminaria japonica OR kelp OR kombu OR kunbu<br>#2. Metabolic OR Reaven OR Syndrome X<br>#3. Cardiovascular OR hypertension OR HTN OR blood pressure<br>#4. Glucose OR diabetes OR hyperglycemia OR Insulin resistance<br>#5. Triglyceride OR HDL OR Cholesterol OR dyslipidemia<br>#6. Waist circumference OR fat OR overweight OR body weight OR obes*<br>#7. #2 OR #3 OR #4 OR #5 OR #6<br>(Metabolic OR Reaven OR Syndrome X OR Cardiovascular OR hypertension OR HTN OR blood pressure OR Glucose OR diabetes OR hyperglycemia OR Insulin resistance OR Triglyceride OR HDL OR Cholesterol OR dyslipidemia OR Waist circumference OR fat OR overweight OR body weight OR obes*)<br>#8. #1 AND #7	2021.09.15	18
